# Physiotherapy protocol to reduce the evolution time of axillary web syndrome in women post-breast cancer surgery: a randomized clinical trial

**DOI:** 10.1007/s00520-025-09373-1

**Published:** 2025-03-28

**Authors:** Jesús Baltasar González-Rubino, Rocío Martín-Valero, María Jesús Vinolo-Gil

**Affiliations:** 1 Department of Physiotherapy, Faculty of Health Sciences, University of Malaga, CTS-1071 Research Group, Malaga, Spain; 2 Rehabilitation Clinical Management Unit, Hospital Punta Europa, Campo de Gibraltar Oeste Health District, 11202 Algeciras, Cadiz, Spain; 3 Department of Nursing and Physiotherapy, University of Cadiz, 11009 Cadiz, Spain; 4 Rehabilitation Clinical Management Unit, Interlevels-Intercenters Hospital Puerta del Mar, Hospital Puerto Real, Cadiz Bay-La Janda Health District, 11006 Cadiz, Spain; 5 Department Biomedical Research and Innovation Institute of Cadiz (Inibica), Research Unit, Puerta del Mar University Hospital, University of Cadiz, 11009 Cadiz, Spain

**Keywords:** Axillary Web Syndrome, Breast Cancer, Physiotherapy, Stretching, Lymphatic System, Physical Therapy

## Abstract

**Objective:**

To reduce the evolution time of axillary web syndrome in women who have undergone breast cancer surgery.

**Methods:**

A prospective, randomized, single-blind clinical trial was conducted on 46 post breast cancer surgery patients from October 2021 to September 2024, in a single university hospital with painful Axillary Web Syndrome (AWS) that restricts arm mobility. The treatment for the intervention group consisted of stretching combined with manual therapy and scar massage to release adhesion and lymphatic cord during 15 physiotherapy sessions of 30 min duration each. The main outcome measures were: healing time, pain, evaluated with Visual Analogue Scaler (VAS) and arm Range of Motion (ROM), evaluated with goniometry.

**Results:**

Significant differences were detected in pain and in ROM. The effect of the intervention varied over time with 95% confidence interval (risk alpha 0.05) and a statistical power of 90% (risk beta 0.1). Comparisons between Control and Intervention Groups showed significant statistical and clinical differences in favour of Intervention Group after 30, 60 and 90 days of intervention at follow-ups for all measured parameters. The proportion of healed patients was significantly higher in the intervention group from day 30 onwards (two-sample test for equality of proportions: *p* < 0.001), indicating a faster recovery in the intervention group.

**Conclusion:**

The results suggested that stretching combined with scar massage and manipulative tissue release techniques lead to a faster recovery and reduce the evolution time of axillary web syndrome. The physiotherapy technique described in this article could be the technique of choice for this surgical sequela.

*Trial Registration:* ClinicalTrials.gov Registry (NCT05115799) on June 10th 2021 and the approval of the Andalucía Ethics Committee (PEIBA code 1909-N1-21, reg. number 171.21).

## Introduction

Breast cancer is the most frequently diagnosed cancer in women worldwide with more than 2 million new cases in 2020 [1].

Axillary Web Syndrome (AWS) was described as a visible and palpable network of cords in the skin of the axillary cavity, tensed by shoulder abduction following surgery for breast cancer, significantly, limiting the function of the ipsilateral upper limb (UL) and causing pain [2–4]. It is also known as “cording”, "axillary string”, “vascular string”, “lymphatic cord”, ‘‘fibrous banding,’’ or ‘‘Mondor’s disease’’ [2, 5].

The frequency of AWS is highly variable. Yeung's systematic review reports ranges from 0.6% to 85.4% after surgical intervention for breast cancer [6]. AWS is an early complication of axillary surgery for breast cancer, which is more common than infection, seroma, or lymphedema [7].

AWS causes pain, reduces shoulder mobility and functionality, and involves a decrease in quality of life [3, 8]. These may affect radiotherapy treatment, leading to delays or even loss of treatment [7, 9, 10]. If sustained over time, it can lead to shoulder rotator cuff disease, adhesive capsulitis, and myofascial pain syndrome[11].

Axillary cords are always present in the axilla and may extend down into the medial ipsilateral arm. These cords frequently extend across the antecubital fossa and into the forearm. They occasionally may extend to the radial aspect of the wrist and into the base of the thumb [10].

AWS usually appears between weeks 8–12 after surgery. It usually resolves by itself within 3–4 months after surgery [6–8], but there is evidence of multiple cases persisting up to 24 months [12, 13]. There are also cases of late recurrence after disappearance [7].

Most current studies point out that the underlying histology of AWS was due to thrombosed lymphatic vessels [12, 14, 15].

Diagnosis of AWS is based on palpation, visual inspection and reported symptoms. Sometimes it is not easy to palpate or see due to a large amount of adipose tissue [6]. Baggi et al. suggested an accurate diagnostic method to improve the diagnosis of AWS [11]. Nuclear magnetic resonance and ultrasound, as well as other methods that may be valid for diagnosis, can be considered [4]. Some studies have used ultrasound to assess the thickness of AWS and its disorganization [16]. Nevertheless, lymphoscintigraphy is currently considered the best method for lymphatic accurate diagnosis [5].

There is currently no treatment of choice for AWS. Physical therapy helps to improve symptoms and outcome, but there is no specific treatment [15]. There is a wide heterogeneity of treatments within physiotherapy [3]. Some studies use stretching together with snapping manual maneuver as a treatment strategy [13], other studies use strength and endurance exercises [17], while other studies use myofascial release [4, 16]. Also, other publications describe exercises combined with manual lymphatic drainage [7, 8, 15]. Systematic reviews and narrative reviews conclude that there is a need for more quality randomized clinical trials to define a treatment of choice for AWS [2, 13, 18].

In addition, physical therapy applied from the early postoperative period could prevent surgical sequelae. So far, no complications have been described [16, 19, 20].

The main objective is to reduce the evolution time of AWS in women who have undergone breast cancer surgery.

## Metodology

### Study design and ethical considerations

**Fig. 1:**
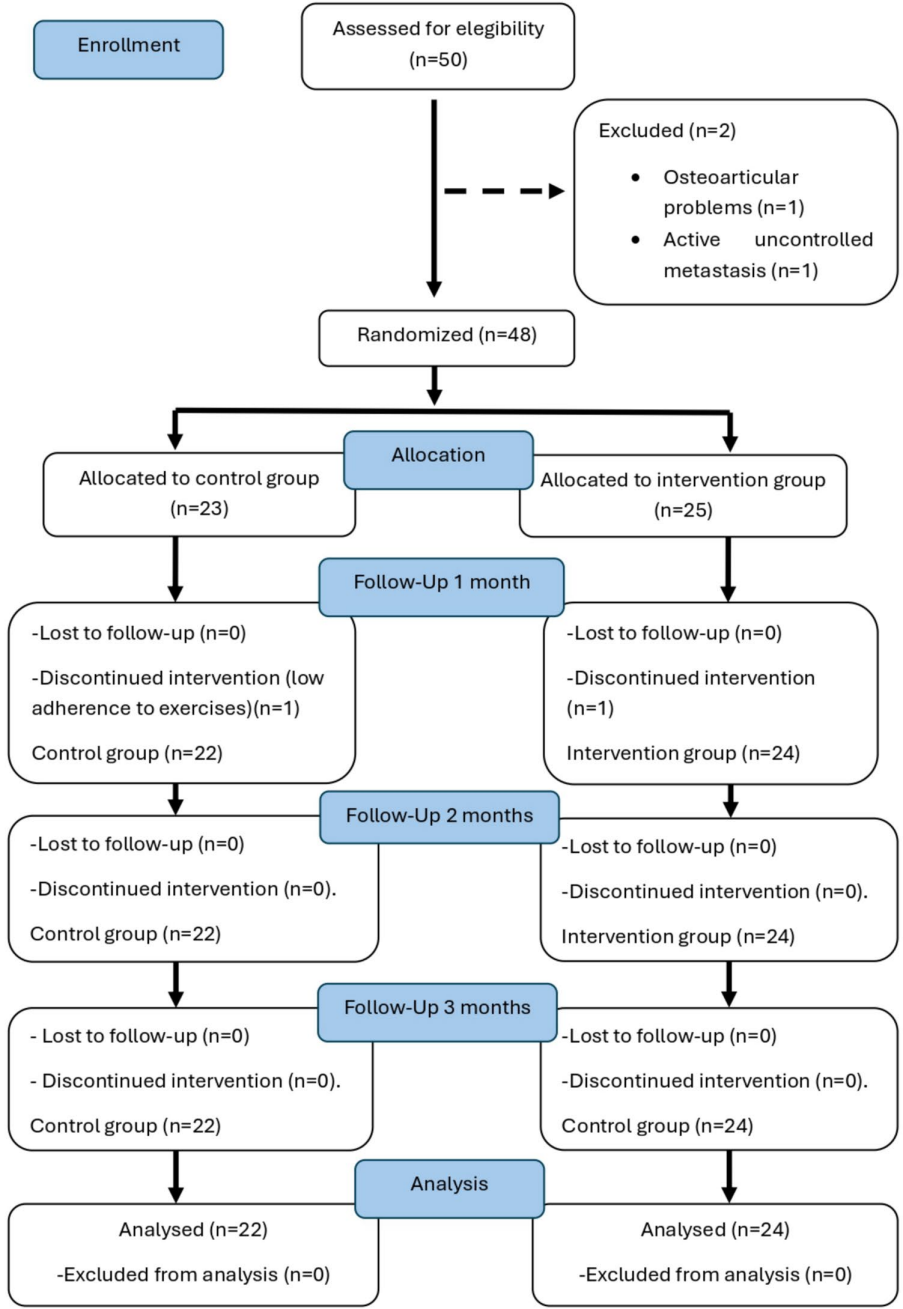
Consort flow chart

This is a two-arm randomized clinical trial. This research uses the guidelines on Standards for Quality Improvement and Excellence in Reporting and Consolidated Standards of Reporting Trials (CONSORT) [21]. Recommendations for Interventional Trials checklist is provided in the Fig. 1. The research procedure was approved by the Andalusian Ethics Committee on Human Research (PEIBA code 1909-N1-21, registration number 171.21) and with Clinical Trial Registration number: ClinicalTrials.gov Registry (NTC05115799). The study was conducted in compliance with the Declaration of Helsinki [22].

### Participants and settings

Inclusion Criteria were as follow: Women over 18 years of age, who underwent breast cancer surgery and AWS appeared within one year after such surgery. Patients who stated that AWS is palpable, caused pain greater than 6 Visual Analogue Scale (VAS) and limits Range of Motion (ROM) (at least with a limitation that does not allow them to raise over 120º of shoulder flexion or 120º of shoulder abduction). Pain was assessed with the patient placed in the supine position at maximum ROM of the shoulder [6]. All users were patients within Campo de Gibraltar West Health Management Area.

Exclusion criteria were as follow: (patients with one or more of the following criteria) Patients with psychological or neurological disorders, with active metastases without chemotherapy treatment, rheumatological or osteoarticular problems limiting joint mobility and to receive a different physiotherapy treatment for AWS than the one prescribed in the study.

Removal criteria were as follow: A mild adverse event; a serious, unexpected or clinically relevant adverse event or not achieving the suggested exercises at least six days a week.

### Sample size

To determine the sample size for comparing two means, we aimed to detect a clinically meaningful difference in healing time for AWS as the primary outcome, measured in days, between intervention and control groups. Both current literature and expert input agreed that a difference of at least 15 days would be clinically relevant. Given that healing time is a quantitative variable, we chose a comparison of means between the two groups as the basis for the sample size calculation.

For this study, we set a confidence level of 95% (alpha = 0.05) and a statistical power of 90% (beta = 0.1), while accounting for an anticipated loss to follow-up of 20%. Based on a standard deviation of 14 days in healing time for standard treatments, we used the pwr package in R to calculate the necessary sample size. To detect a difference of 15 days or more between groups with the specified confidence and power, 18 subjects per group were required. Adjusting for the potential 20% follow-up loss, the total sample size needed was determined to be 46 participants.

Randomization allocation: Before commencing the study, Excel program ‘randomization’ tool was used to provide an allocation sequence list for patients from 1 to 46. The position on the list would be assigned on the arrival order. As patients arrived at the clinic for the first evaluation, they were informed about the study. If they agreed to participate, and after giving written consent, their physiotherapist would enroll the patient to either control or intervention group, according to the previously mentioned Excel list.

Masking: Blinding could not be performed due to the nature of the intervention. Physiotherapy studies often face challenges in masking both the intervention and the therapist.

### Intervention

-**Control group** Users are trained by the physiotherapist in the first consultation on Codman's pendulum exercises and on self-assisted stretching in seated and standing positions. The stretches are explained to the patient by the specialist physiotherapist and initially performed together to correct possible errors. Advice is given on possible postural compensations. The importance of performing these exercises daily is explained. At least 15 repetitions of each exercise holding the stretch for 15–20 s. The exercises are described in Appendix 1.

After diagnosis, patients attend group therapy twice where exercises are repeated and corrected. The groups are limited to less than 6 people to ensure quality care. In addition, an informative talk about lymphoedema and AWS is delivered, in which preventive measures (usual care) are specified. Follow up is carried out monthly for the next 3 months. At each review, adherence to the program is assessed, confirming that the user performs the exercises at least 6 days a week. In the case any patient in the control group received additional treatment for AWS, this has to be notified to the specialist physiotherapist who may consider the exclusion from the study.

**-Intervention group**: Users receive the same training as control group patients, but intervention group patients receive 15 physiotherapy sessions within three weeks, that is 5 sessions per week delivered by the same specialist physiotherapist. Each session lasts 30 min. During the sessions, the patient is placed on the couch in the supine position. The physiotherapist applies stretches to the affected arm, considering patient's tolerance, the pain never beyond level 6 in the VAS. Initial warm-up is performed with self-assisted mobility of the upper limb and bringing the arm to the maximum flexion or abduction that the patient can tolerate without the appearance of elevated pain. The elbow is usually in almost full extension (if the cord allows such a position), supination, wrist extension and thumb opposition. For no more than 30 s in this position, the physiotherapist palpates the cord and works through it, with friction similar to the scar massage maneuver at the level of the axillary or mammary scar, where the AWS usually originates. This manipulative technique of friction is also applied to the cord in the area which the patient describes as discomforting. The friction is carried out perpendicular and longitudinally to the cord. Longitudinal manipulations are more frequent than perpendicular and they are based on pulling the skin distally and in the areas where palpation of the cord is most uncomfortable for the patient. The maneuver should be gentle to avoid the appearance of skin erythema. Out of the 30 min for each session, the first 5 min are dedicated to warm-up performing pendular exercises. As soon as warm up is completed, the patient is placed in the supine position and the exercises described in Appendix 2 are performed. For the next 25 min the patient assisted by the specialist physiotherapist will perform stretches described in Appendix 2 in 30 s slots. Meanwhile, the specialist physiotherapist palpates and performs the aforementioned described maneuvers.

### Outcomes

Firstly, the user's administrative data is collected: such as age, marital status, employment status, or educational level. Also, whether she has ever become a mother and if so, when. Regarding lifestyle, whether she practices sport and how often. whether she lives in an urban or rural area. It is also considered whether she smokes or not. Regarding medical records, body mass index (BMI), type of tumor, when the axillary web syndrome appeared, number of lymph nodes removed, whether her surgery was radical or conservative, whether she has received radiotherapy, and finally whether the patient received breast reconstruction or not. The assessment for all the outcomes was carried out by the same specialist physiotherapist.

- Primary Outcome:
  Healing time: it is the time frame expressed in days for the resolution of AWS symptoms [6]. If such resolution is not achieved, at least at maximum ROM of the shoulder with less than 6 points on the VAS score and shoulder flexion and/or abduction greater than 120º.
- Secondary Outcomes:
  Range of motion (ROM): For the assessment of the mobility goniometry has been used. Goniometer is the standard instrument for measuring the ROM. Patients were asked to move their arms in flexion, extension, abduction and external and internal rotation of the shoulder. The goniometric measurement was taken with the patient in the supine position, close to the edge of the examination table. The specialist physiotherapist checked that there were no postural compensations. It was considered that the maximum ROM for the flexion and abduction was 180º, for extension it was 45º, 100º for internal rotation and 80º for external rotation. Finally, a single index was calculated as the percentage of global movement [23].
  Visual Analog Pain Scale (VAS): According to the National Cancer Institute (NIH), it is a tool used to help the professional assess the intensity of certain sensations and feelings, such as pain. The Visual Analog Scale for pain is composed of a straight line on which an extreme means no pain and the other extreme means the worst pain imaginable. Extreme pain corresponds to 10 points. No pain corresponds to 0 points.
  The patient marks a point on the line that matches the amount of pain they feel. Also known as VAS [24].

### Statistical analysis

Descriptive statistics were computed as means and standard deviations for continuous variables, and medians with interquartile ranges for non-normally distributed data. Proportions and 95% confidence intervals were calculated for categorical variables. Cohort homogeneity at baseline was assessed by comparing all measured variables between patient groups using nonparametric Wilcoxon tests. P-values were adjusted using the FDR method to control for type I error.

To assess healing time, a two-sample test for equality of proportions was conducted at baseline, and on the 30th, 60th, and 90th days. These analyses compared the proportion of patients who had healed (defined as those with a VAS score of less than 6 points and shoulder flexion and/or abduction greater than 120º during follow-up) between the control and intervention groups.

To evaluate the effect of physiotherapeutic treatment on the pain and ROM of breast cancer surgery patients, mixed-effects regression models were employed. These models accounted for both fixed effects (e.g., intervention group, time, and covariates such as age, BMI, and tumor stage) and random effects (individual variability). This approach controlled for baseline differences and allowed for a more accurate estimation of treatment effects. Models were adjusted for several outcome measures, including the visual analog scale (VAS), and interactions between the intervention group and time were included. Model fitting followed Zuur & Ieno’s protocol [25],with random effects for patient identity and fixed effects selected via backwards elimination. Model comparison was conducted using corrected Akaike.

Information Criterion (AICc), and the significance of categorical factors was assessed via ANOVA, with post-hoc comparisons using the emmeans package. Assumptions were verified through residual analysis and Shapiro–Wilk (normality) and Levene (homoscedasticity) tests.

Changes in goniometry were analyzed with two-way ANOVAs, assessing group (control/intervention) and time as fixed factors, including their interaction.

All analyses were performed using R v.4.4.2 [26]. Statistical significance was set at α = 0.05.

#### Assessment procedure (Adherence monitoring):

If during regular post-surgery checkups AWS was diagnosed, the patient was recruited for the study after signing informed consent. Patients were requested to answer a clinical interview in person on day one, day thirty, sixty and on day ninety as aforementioned in the outcomes section. Performance and adherence were assessed from day 30 onwards.

In person appointment was reminded via telephone a few days beforehand. In case the patient could not attend, a three-day window was considered.

On appointment day, the specialist physiotherapist showed the patient how to perform the exercises and checked that the patient was able to perform them correctly.

Patients were required to perform those exercises at home at least six times per week. When a patient was unable to do so, she was excluded from the study.

A paper booklet and a YouTube video were provided to help them perform exercises accordingly. When needed, telephone and walk-in advice was offered.

Informed consent statement: An informed consent form was prepared, which had to be signed by all the subjects participating in the study who previously received sufficient information about the objectives and the procedure of the study. They were also informed of the possibility of revoking the consent given at any time without having to justify their decision without prejudice. All necessary permits were requested from the institutions for the development of the research.

## Results

### Recruitment and characteristics of participants

Patients attending the Lymphoedema Unit with clinical manifestations of AWS (pain of more than 6 points on the VAS scale and reduced shoulder mobility of less than 120º of flexion and/or abduction). Enrollment began in October 2021 until September 2024. 46 patients participated, 24 in the intervention group and 22 in the control group.

The bio-demographic data of all participants at baseline showed homogeneity and non-significant differences between the two groups in terms of age, weight, height, body mass index, type of oncological treatment, type of surgical treatment, stage and type of cancer (Table 1). No type 1 error could be detected.

**Table 1:** Baseline bio-demographic and clinical characteristics of study participants

Outcome	Levels	Control	Intervention	p.value	p.adjust	sig
Age		49.09 ± 8.7	50.54 ± 10.61	0.817	0.984	
Marital Status	Single	9.09% (a)	29.17% (ab)	0.293	0.688	
	Married/ Civil partnership	72.73% (b)	50% (a)			
	Divorced/separated	13.64% (a)	16.67% (ab)			
	Widowed	4.55% (a)	4.17% (b)			
Level Completed Studies	No education	4.55% (a)	0% (a)	0.068	0.447	
	Primary	13.64% (a)	20.83% (ab)			
	Secondary	63.64% (b)	33.33% (b)			
	University	18.18% (a)	45.83% (b)			
Residence	Rural areal	0% (a)	4.17% (a)	1	1	
	Urban area	100% (b)	95.83% (b)			
Children	No	9.09% (a)	29.17% (a)	0.139	0.518	
	Yes	90.91% (b)	70.83% (b)			
Mother over 30 years	No	72.73% (a)	54.17% (a)	0.233	0.632	
	Yes	27.27% (b)	45.83% (a)			
Smoker	No	68.18% (a)	58.33% (a)	0.185	0.594	
	Yes	9.09% (b)	29.17% (ab)			
	Ex-smoker	22.73% (b)	12.5% (b)			
Weight (Kg)		62.07 ± 10.97 (a)	58.87 ± 8.25 (b)	0.153	0.547	
Size (cm)		1.59 ± 0.06	1.62 ± 0.06	0.058	0.426	
BMI		24.63 ± 4.53	22.31 ± 2.82	0.08	0.473	
Employment Status	Active	80% (a)	71.43% (a)	0.386	0.714	
	Unemployed	20% (b)	14.29% (b)			
	Retired	0% (b)	14.29% (b)			
Sporting activities	No	40.91% (a)	29.17% (a)	0.538	0.82	
	Yes	59.09% (a)	70.83% (b)			
Type of cancer	Ductal	95% (a)	91.67% (a)	1	1	
	Lobulillar	5% (b)	4.17% (b)			
	In situ	0% (b)	4.17% (b)			
Number of nodes removed		4.45 ± 4.43 (a)	4.79 ± 4.8 (b)	0.63	0.874	
Type of Surgery	Conservative	68.18% (a)	70.83% (a)	1	1	
	Radical	31.82% (b)	29.17% (b)			
Breast Reconstruction	No	81.82% (a)	75% (a)	0.725	0.928	
	Prótesis	18.18% (b)	25% (b)			
Radiotherapy	No	5% (a)	13.04% (a)	0.61	0.859	
	Yes	95% (b)	86.96% (b)			
No. of sessions Radiotherapy		13 ± 5.71 (a)	12.5 ± 4.93 (b)	0.51	0.79	
Chemotherapy	No	23.81% (a)	41.67% (a)	0.342	0.708	
	Yes	76.19% (b)	58.33% (a)			
No. of cycles Chemotherapy		14.56 ± 3.76 (a)	14.79 ± 4.37 (b)	0.552	0.828	
Monoclonal antibodies	No	71.43% (a)	65.22% (a)	0.752	0.949	
	Yes	28.57% (b)	34.78% (a)			
Hormone therapy	No	28.57% (a)	13.04% (a)	0.272	0.663	
	Yes	71.43% (b)	86.96% (b)			
Cancer Stage (TNM scale) T	T1	54.55% (a)	54.17% (a)	0.772	0.965	
	T2	36.36% (a)	29.17% (ab)			
	T3	4.55% (b)	12.5% (bc)			
	T4	4.55% (b)	0% (c)			
	Tx	0% (b)	4.17% (bc)			
Stage Cancer (TNM scale) N	N0	50% (a)	50% (a)	0.801	0.97	
	N1	36.36% (a)	45.83% (a)			
	N2	4.55% (b)	4.17% (b)			
	N3	4.55% (b)	0% (b)			
	Nx	4.55% (b)	0% (b)			
Stage Cancer (TNM scale) M	M0	45.45% (a)	50% (a)	0.777	0.65	
	Mx	54.55% (a)	50% (a)			
Time since diagnosis (months)		0.82 ± 1.84 (a)	1.58 ± 3.02 (a)	0.219	0.615	
Time from surgery to appearance of Lymphatic Thrombus (days)		38.64 ± 58.38	60.92 ± 99.59	0.791	0.965	
Dominance	Right handed	95.45% (a)	91.67% (a)	1	1	
	Left handed	4.55% (b)	8.33% (b)			
Days per week exercise		6.86 ± 0.35 (a)	6.75 ± 0.44 (b)	0.345	0.708	

### Effectiveness of the intervention on the healing time outcome

**Fig. 2:**
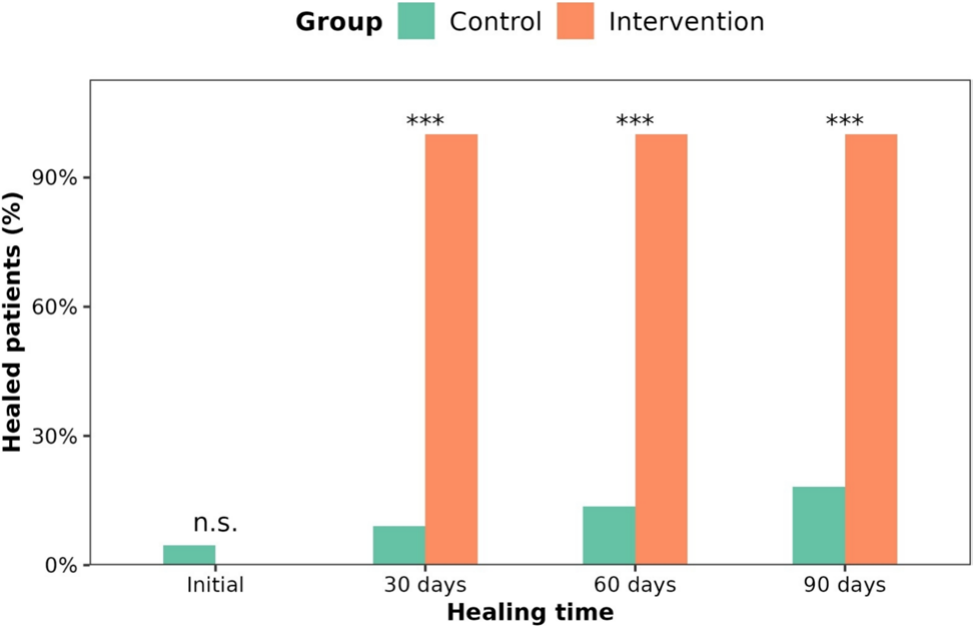
Percentage of patients healed for control group and intervention group at 30th day, 60th day and 90.^th^ day. (n.s.: not significant)

By the 30th day, all patients in the intervention group had healed, as evidenced by VAS scores of less than 6 points and shoulder flexion and/or abduction greater than 120º. In contrast, fewer than 20% of patients in the control group had healed by the 90th day. Thus, the proportion of healed patients was significantly higher in the intervention group from day 30 onwards (two-sample test for equality of proportions: *p* < 0.001; Fig. 2), indicating a faster recovery in the intervention group.

### Effectiveness of the intervention on the VAS outcome

**Fig. 3:**
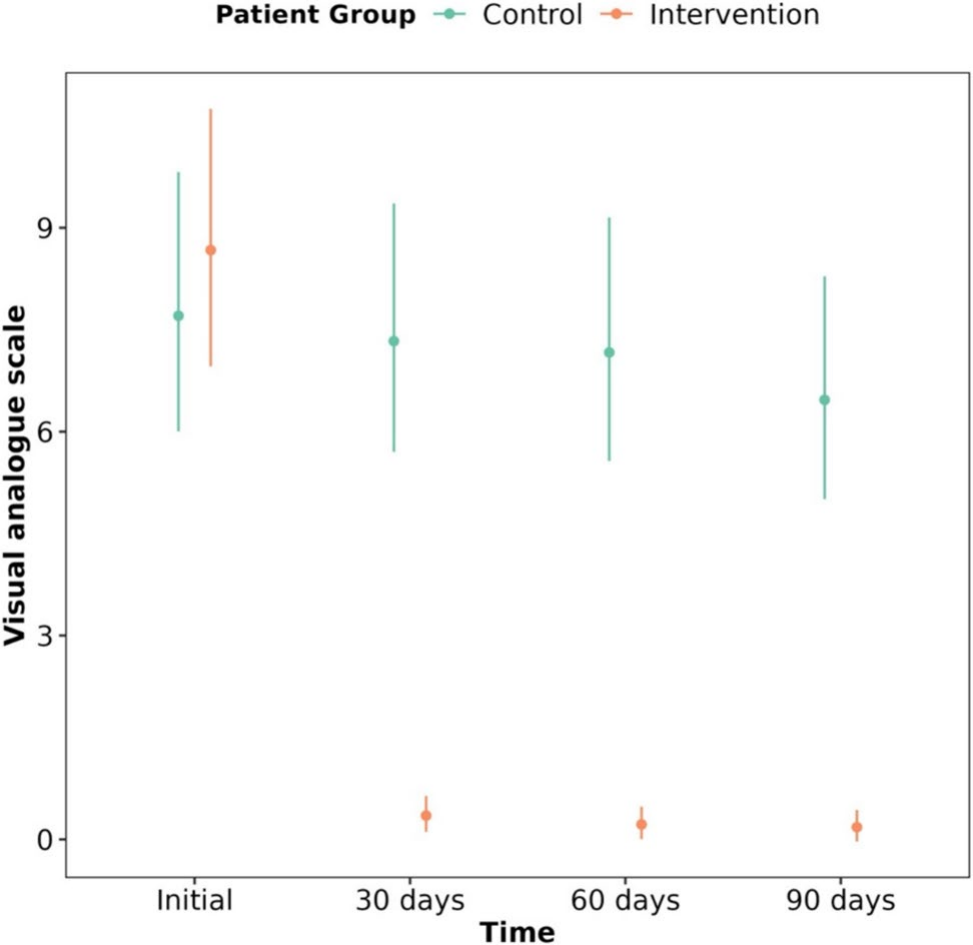
VAS score evolution for control group and intervention group and baseline, at 30th day, 60th day and 90th day

There is a significant association between the total VAS score and the time of measurement of the test depending on the study group. On one hand, patients in the intervention group obtain a significant decrease in VAS score reaching pain levels very close to zero from the 30th day (*P* < 0.001), maintaining this improvement through the remaining visits (Fig. 3; Table 2). On the other hand, patients in the control group improve much gradually, never reaching the scores of the intervention group. In fact, in the control group, a significant improvement is only detected in the 90th day compared to the initial moment (Table 2).

**Table 2:** VAS score evolution for control group and intervention group and baseline, at 30th day, 60th day and 90th day

**Group**	**Measurement moment**	**EMM [CI 95%]**		**Group**	**Contrast**	**Difference**	**z ratio**	**p value**	
control	initial	8.71 ± 0.97 [7.01—10.83]		control	initial / 30 days	1.04 ± 0.06	0.79	0.859	
control	30 days	8.34 ± 0.93 [6.71—10.36]		control	initial / 60 days	1.07 ± 0.06	1.15	0.658	
control	60 days	8.17 ± 0.91 [6.57—10.16]		control	initial / 90 days	1.17 ± 0.06	2.75	0.03	*
control	90 days	7.47 ± 0.83 [6.01—9.29]		control	30 days / 60 days	1.02 ± 0.06	0.36	0.984	
intervention	initial	9.67 ± 0.96 [7.96—11.74]		control	30 days / 90 days	1.12 ± 0.06	1.97	0.201	
intervention	30 days	1.35 ± 0.13 [1.11—1.64]		control	60 days / 90 days	1.09 ± 0.06	1.61	0.375	
intervention	60 days	1.22 ± 0.12 [1—1.48]		intervention	initial / 30 days	7.17 ± 0.39	36.53	0.0e + 00	***
intervention	90 days	1.18 ± 0.12 [0.97—1.43]		intervention	initial / 60 days	7.93 ± 0.43	38.56	0.0e + 00	***
				intervention	initial / 90 days	8.2 ± 0.44	39.23	0.0e + 00	***
				intervention	30 days / 60 days	1.11 ± 0.06	1.87	0.239	
				intervention	30 days / 90 days	1.14 ± 0.06	2.51	0.059	
				intervention	60 days / 90 days	1.03 ± 0.06	0.063	0.921	

### Effectiveness of the intervention on the ROM outcome

**Fig. 4:**
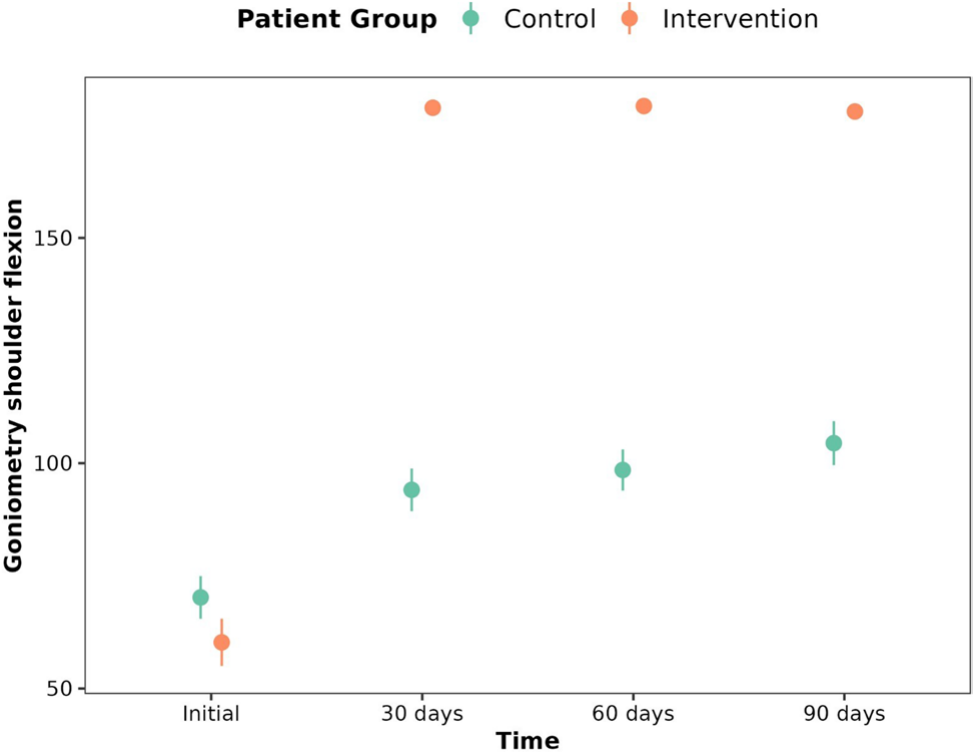
Evolution of shoulder flexion for control group and intervention group at 30th day, 60th day and 90th day

**Fig. 5:**
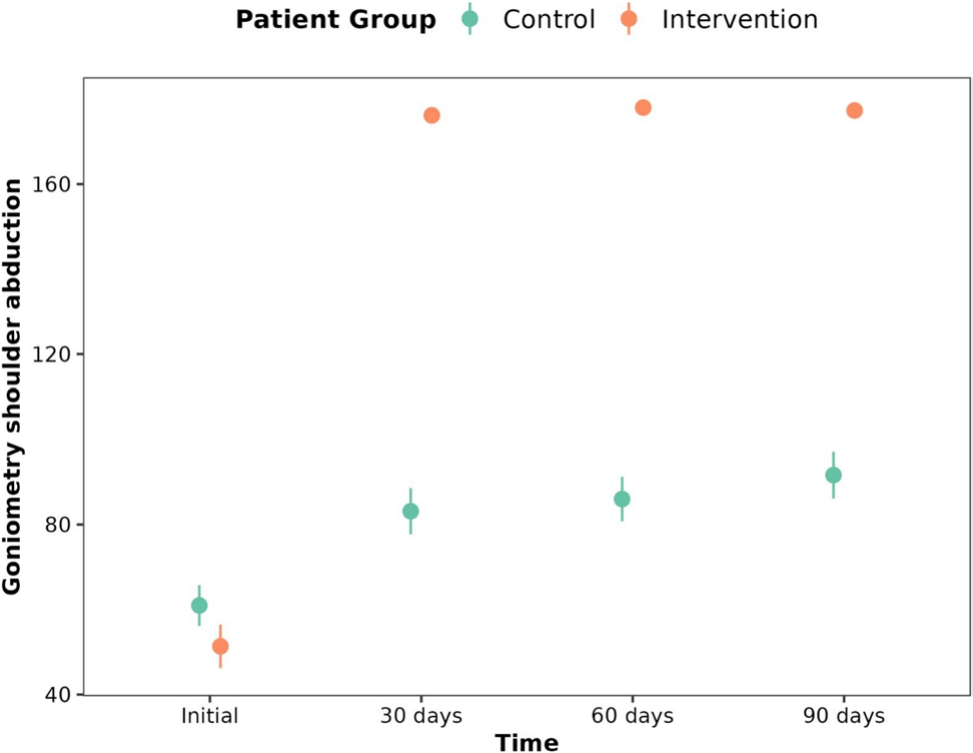
Evolution of shoulder abduction for control group and intervention group at 30th day, 60th day and 90th day

In the case of the variables flexion (*p* < 0.001) (Fig. 4), abduction (*p* < 0.001) (Fig. 5), extension (*p* < 0.001), adduction (*p* < 0.001), external (*p* < 0.001) and internal rotation (*p* < 0.001) of the shoulder, the control patients gradually gained an increase in flexion over time, while the intervention patients reached a much greater increase from the 30th day and maintained this flexion through all visits. This is also the case for the outcomes flexion (*p* < 0.001), supination (*p* < 0.001) and pronation (*p* < 0.001) of the elbow and for radial (*p* < 0.001) and ulnar deviation (*p* < 0.01) of the wrist (Table 3).

**Table 3:** ROM of the affected upper limb for control group and intervention group, at 30th day, 60th day and 90th day

			**Initial**	**30 days**	**60 days**	**90 days**	**p-value**	
Shoulder	Flexion	Control	70.23 ± 22.18	94.09 ± 22.25	98.5 ± 21.41	104.45 ± 22.92	1.2E-29	***
	Flexion	Intervention	60.26 ± 25.15	178.87 ± 1.94	179.26 ± 1.29	178.04 ± 5.64		
	Extension	Control	26.41 ± 9.29	36.95 ± 10.65	38.23 ± 10.6	40.36 ± 10.45	1.4E-09	***
	Extension	Intervention	25.39 ± 11.87	57.52 ± 3.23	58.3 ± 2.32	58.52 ± 2.41		
	Adduction	Control	9.5 ± 2.35	11.14 ± 2.08	11.77 ± 1.97	11.95 ± 1.89	2.8E-11	***
	Adduction	Intervention	8.39 ± 1.85	14.7 ± 0.47	14.87 ± 0.34	14.87 ± 0.34		
	Abduction	Control	60.95 ± 22.45	83.14 ± 25.47	86 ± 24.62	91.64 ± 25.8	7.6E-31	***
	Abduction	Intervention	51.35 ± 24.53	176.17 ± 3.23	178 ± 2.13	177.3 ± 5.77		
	External Rotation	Control	27.73 ± 14.67	35.68 ± 13.83	38.55 ± 16.86	40.27 ± 17.3	6.4E-26	***
	External Rotation	Intervention	23.96 ± 11.26	87.09 ± 2.54	88.35 ± 1.97	88.17 ± 2.55		
	Internal Rotation	Control	25.95 ± 10.21	33.95 ± 11	36.86 ± 14.66	39.45 ± 14.33	1.2E-20	***
	Internal Rotation	Intervention	20.61 ± 8.93	67.7 ± 2.82	69.04 ± 2.12	68.74 ± 2.78		
Elbow	Extension	Control	−0.23 ± 1.85	0 ± 0	−0.23 ± 1.07	0 ± 0	0.016	*
	Extension	Intervention	−2.04 ± 4.43	0.09 ± 0.42	0.09 ± 0.42	0.09 ± 0.42		
	Flexion	Control	112.95 ± 4.89	116.64 ± 3.61	117.64 ± 4.18	117.77 ± 3.6	0.000012	***
	Flexion	Intervention	107.3 ± 8.24	119 ± 1.31	119.3 ± 1.33	119.48 ± 1.53		
	Pronation	Control	66.32 ± 4.52	70.95 ± 3.08	72.41 ± 3.42	71.68 ± 2.85	4.9E-07	***
	Pronation	Intervention	59.35 ± 7.82	72.43 ± 2.43	73.04 ± 1.92	73 ± 2.47		
	Supination	Control	79.36 ± 6.43	84.32 ± 5.92	86 ± 5.77	86.32 ± 5.34	2.2E-07	***
	Supination	Intervention	68.48 ± 13.95	87.96 ± 1.8	88.61 ± 1.53	88.52 ± 1.68		
Wrist	Cubital Deviation	Control	30.73 ± 2.33	32 ± 2.2	32.64 ± 1.53	33 ± 1.51	0.009	**
	Cubital Deviation	Intervention	28.09 ± 4.54	32.96 ± 3.25	33.26 ± 2.63	33.17 ± 2.64		
	Radial Deviation	Control	18.77 ± 4.26	18.41 ± 1.18	18.64 ± 1.47	18.73 ± 1.7	2.6E-06	***
	Radial Deviation	Intervention	16.3 ± 1.58	20 ± 1.45	20.09 ± 1.5	20.09 ± 1.59		
	Flexion	Control	67.77 ± 2.81	68.77 ± 2.99	69.5 ± 3.05	69.32 ± 3.37	0.082	
	Flexion	Intervention	63.04 ± 9.22	70.61 ± 8.81	70.65 ± 8.8	69.96 ± 8.64		
	Extension	Control	66.27 ± 3.4	67.64 ± 3.68	68.41 ± 3.61	68.5 ± 3.83	0.089	
	Extension	Intervention	61.57 ± 8.29	69.61 ± 8.87	69.57 ± 8.63	68.61 ± 9.17		

Complications during the treatment: Two of the patients in the intervention group developed a slight thickening around the forearm over the cord. This disappeared spontaneously after a few days.

## Discussion

The main finding of this study was that 22 out of the 24 patients in the intervention group did not suffer from AWS at the end of the treatment. The remaining 2 patients had no pain or limitation of mobility. All patients in the control group still suffered AWS on the 90th day of follow-up, with limited mobility and pain.

The correct performance of the exercises together with adherence are considered key factors in the reliability on this study results. Torres et al. [7] in their study also showed good and parallel adherence in both groups. Klein et al. [20] offered a paper booklet and phone call assessment one week and one month after the intervention. Cho et al. [15] focused on the support for the physical therapy and manual lymphatic drainage group during the first week. This posed a confusing factor in the perception of pain within such group. Their study neither detail the applied exercises nor the intervention group patients´ adherence. Moreover, the study by Cho et al. [15] considered unethical the presence of a control group, disregarding such group for the study. As a result, this study showed a big dropout rate, with a total loss of 29, out of the initial 70 patients within the intervention group.

As aforementioned, some studies had considerable patient drop-outs. The study by Meer et al. started with 36 patients in the intervention group and only 20 remained for the whole period [8], the study by Muñoz et al. where 31 patients in the intervention group initially started.

only 20 finished [19] and the study by Ibrahim et al. where they initially had 59 patients and 33 patients finished at 18 months of follow-up [16]. In contrast, other studies also followed up for more than one year without such a high percentage in drop-outs [17, 27].

Regarding the onset of postmastectomy lymphoedema, treatment of AWS does not appear to promote the development of such sequelae or other adverse effects [8, 13, 20]. Some studies highlighted the benefits of starting physiotherapy early to reduce pain [20] and to improve mobility and strength [19]. Some studies excluded patients with lymphoedema, and state that there is a need for further research to analyze the association between AWS and lymphoedema [7]. The study by Klein et al. had no conclusions regarding the relationship between AWS and lymphoedema, since surgery is currently usually conservative and without axillary emptying, in addition to the fact that postmastectomy lymphoedema usually develops 2 years after the operation. Therefore, studies should have longer follow-ups to draw conclusions in this regard. One study used moist heat as a treatment technique for AWS, but this may facilitate the development of lymphoedema, as it is known that thermal measures may provoke its development [4].

Many publications conclude that there is high heterogeneity of physiotherapy treatments. They indicate that more AWS related research is needed to have a physiotherapy treatment protocol of choice as there is currently none [2, 10, 18, 28]. There are few randomized clinical trials and some of them had small sample sizes such as the study by Datar et al. with N = 10 [4] or the study by Meer et al. with N = 36 [8]. There is also no ideal timing within current treatment approaches [2] and AWS may possibly be under-diagnosed [10]. All publications highlight the need for more research with better designs and larger sample sizes [19]. Collaborative research in the multidisciplinary team with oncologists, surgeons, physiotherapists would be ideal [7, 28]. Also, histological and pathophysiological studies of AWS are needed to better understand the mechanism of production and thus, facilitate a reference physiotherapy treatment [3]. As some studies state, the lymphatic duct undergoing AWS could have future recanalizations or collateralizations and therefore disappear [28]. This theory has been explored using lymphoscintigraphic imaging, although it was a retrospective study, its conclusions would be stronger if the study had been prospective [5]. Current studies link AWS to lymphatic origin, excluding a venous process [3, 5, 9].

In our intervention group, within the 15 physiotherapy sessions, 21 of the 24 patients (87.5%) felt the un-mooring effect. This effect can be described by the specialist physiotherapist as the sudden release of the AWS, comparing it to the release of the ropes when a vessel departs from the quay. An average of 3.62 clicks per patient, with 3 of the patients feeling more than 15 clicks. This effect was also cited in the study by Sandrin et al. and Josehans et al. and in the systematic review by Yeung et al. [6, 13, 29].

### Strengths and Limitations:

- Our study registered good adherence to the scheduled activity in each group with almost no loss of recruited patients in either group. The physiotherapist ensured correct adherence to the exercises.
- Considering the good results in the intervention group in all outcomes from the first follow-up on day 30 after the first examination, this physiotherapy technique could become an effective and quick treatment for AWS.
- Our study was single center, so its external validity may be questioned. It would be highly advisable to perform this study as multi center to be able to reach a wider spectrum of target population.
- Blinding of the intervention could not be carried out due to the nature of the intervention. Physiotherapy studies often face this challenge as it is difficult for the patient to be unaware of the group they belong to.

*Future Directions

Ideally, future studies should include imaging tests, such as Indocyanine Green Lymphography [30] or Lymphoscintigraphic imaging [5], to better understand the process of making AWS disappear. Few studies use imaging techniques that visualize AWS, and out of the few that do, they seem to describe that the cord disappears and new recanalizations and collateralizations appear [5].

## Conclusions

The results suggest that stretching combined with scar massage and manipulative tissue release techniques lead to a better recovery and reduce the evolution time of axillary web syndrome. The physiotherapy technique described in this article could be the technique of choice for this surgical sequela.

## Figures and Tables

**Fig. 6:**
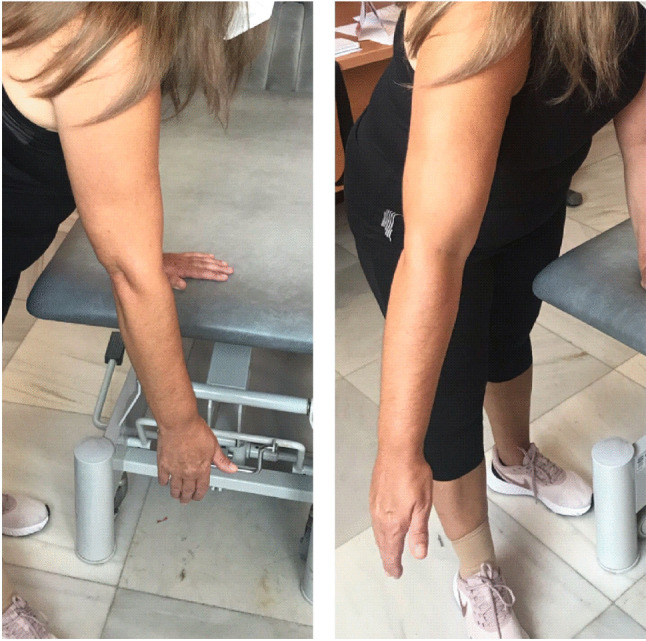
Codman Pendulum exercises

**Fig. 7:**
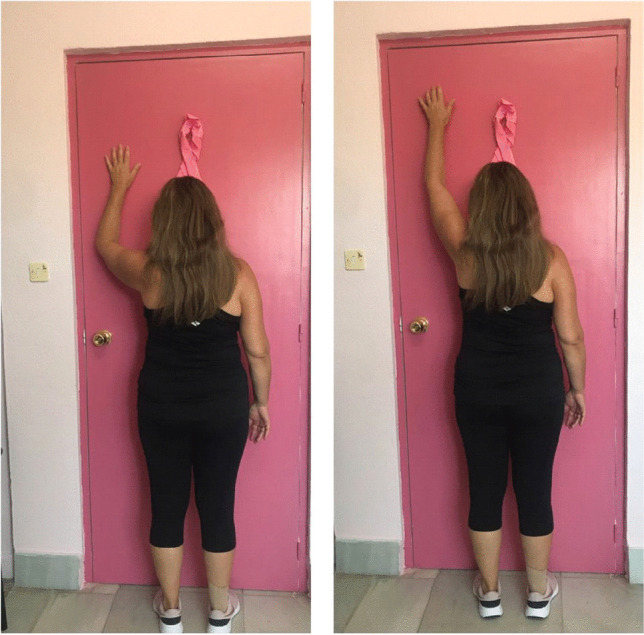
Standing, affected shoulder flex sliding palm of the hand on the wall, up and down. Keeping maximum flexion during 15 s

**Fig. 8:**
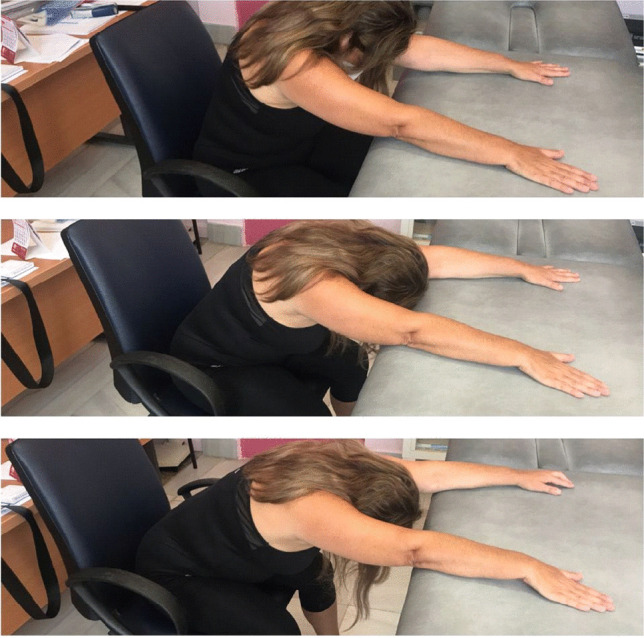
Seated, affected shoulder flexion. Seated on a wheeled chair, leaning elbow on a table. Shoulder flexion sliding chair, backwards and forwards

**Fig. 9:**
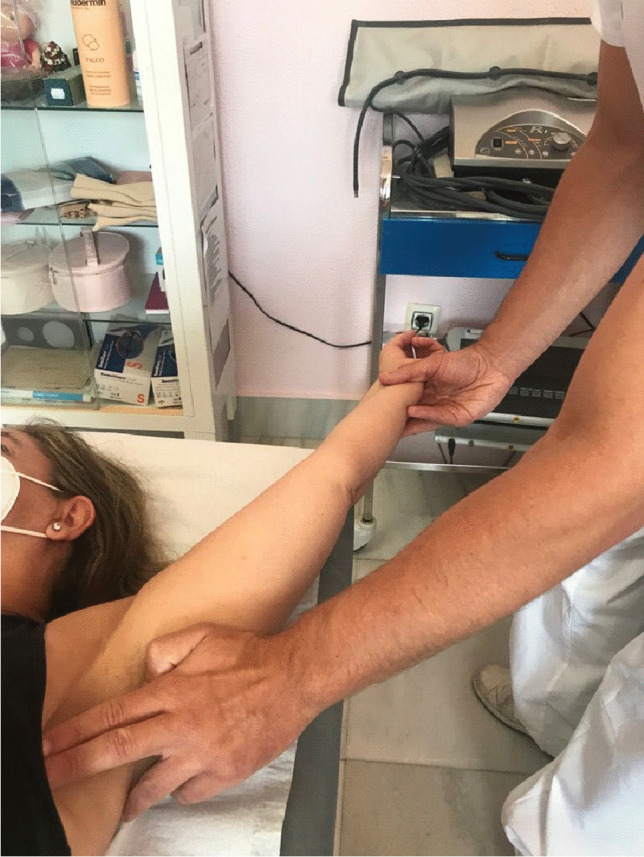
The Physiotherapist rests the affected limb diagonal to regular movement only if pain due to stretching is moderate

**Fig. 10:**
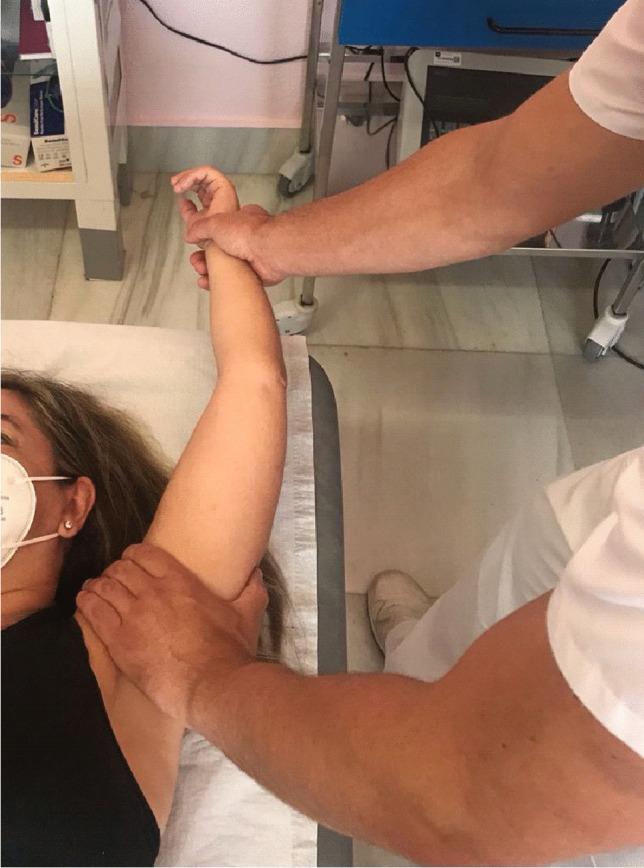
The Physiotherapist rests the limb in shoulder flexion following elbow extension

**Fig. 11:**
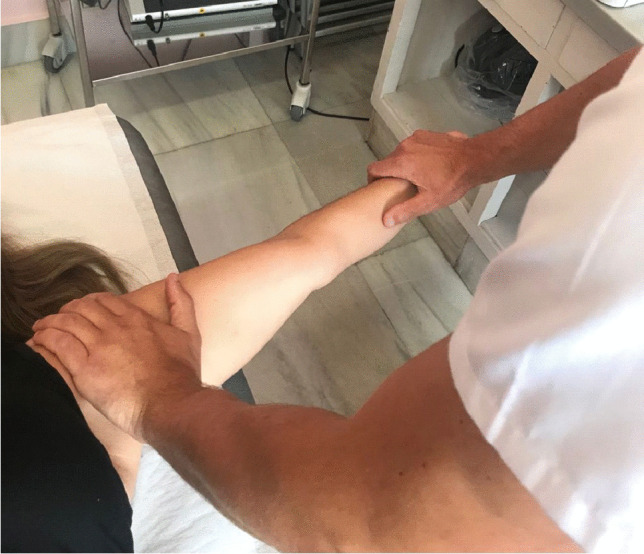
The Physiotherapist places the limb in abduction position while the patient can tolerate discomfort

**Fig. 12:**
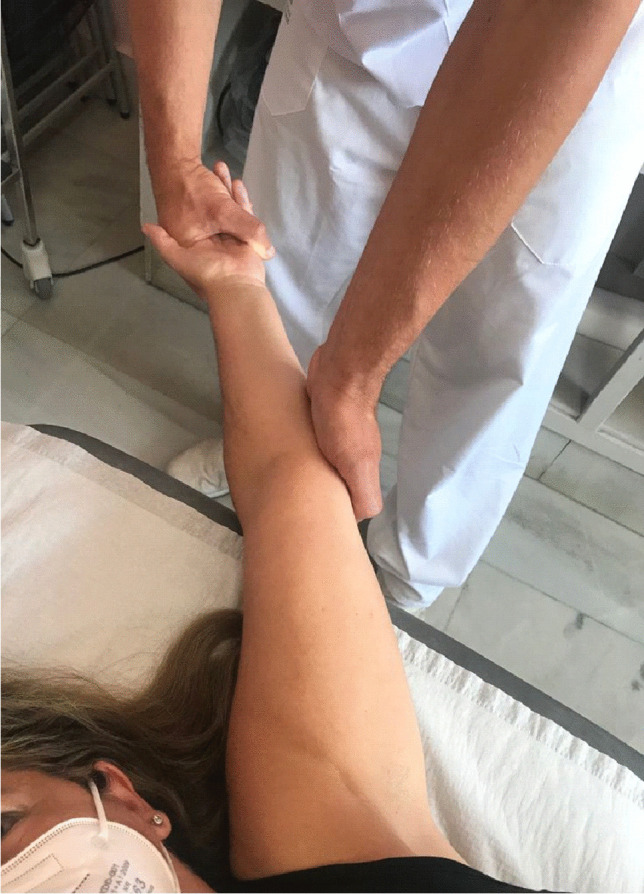
In case the cord reaches the elbow, the Physiotherapist holds the elbow extension combined with supination of the limb. The shoulder will be kept in flexed position as long as pain is moderate to keep the tension of the cord

**Fig. 13:**
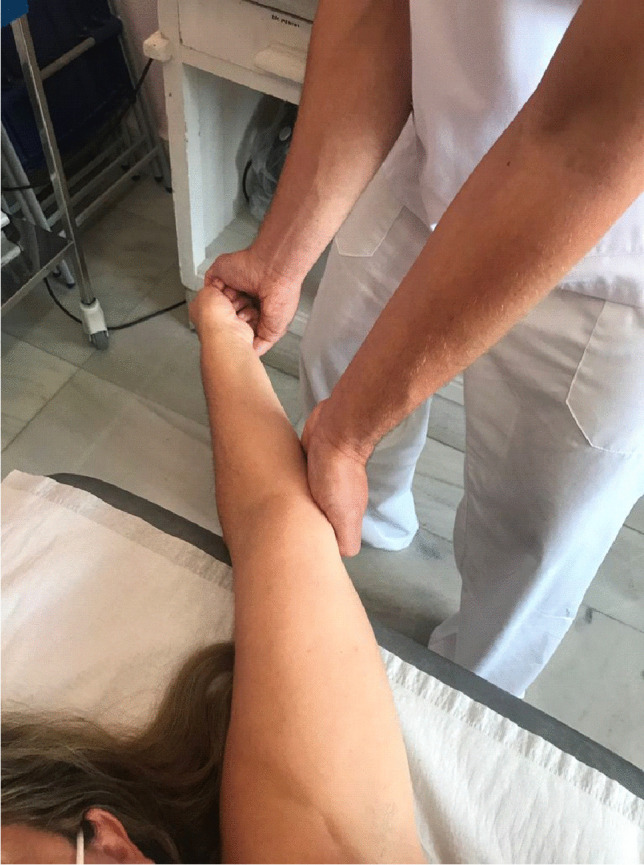
If the thrombus reaches the first finger, the Physiotherapist will try to reach the ulnar deviation of the wrist together with the flexion of the thumb. The shoulder will be kept in a flexed position and the elbow in an extended supination position

## Data Availability

No datasets were generated or analysed during the current study.

## Appendix 1

These exercises are explained to the patient to be executed at home. Figs. 6, 7, 8, 9, 10, 11, 12 and 13

Lymphoedema prevention exercises (please visit our YouTube channel “Unidad Linfedema Algeciras”).

Signed consent was given by the patients for the publication of the photographs in which they appear.

## Appendix 2

Manual Therapy, Scar Massage and exercises for the Intervention Group.

## Declarations

### Trial registration number

Clinical Trials.gov Registry (NTC05115799).

### Protocol

The study protocol was approved by the Andalusia Ethics Committee.
